# Deep conservation complemented by novelty and innovation in the insect eye ground plan

**DOI:** 10.1073/pnas.2416562122

**Published:** 2024-12-30

**Authors:** Ke Gao, Antoine Donati, Julia Ainsworth, Di Wu, Eleanor R. Terner, Michael W. Perry

**Affiliations:** ^a^Department of Cell & Developmental Biology, School of Biological Sciences, University of California San Diego, La Jolla, CA 92093

**Keywords:** evolution, development, visual system, photoreceptor, cell fate

## Abstract

A spectacular diversity of forms and features allow species to thrive in different environments, yet some structures remain relatively unchanged. Insect compound eyes are easily recognizable despite dramatic differences in visual abilities across species. It is unknown whether distant insect species use similar or different mechanisms to pattern their eyes or what types of genetic changes produce diversity of form and function. We find that flies, mosquitos, butterflies, moths, beetles, wasps, honeybees, and crickets use homologous developmental programs to pattern their retinas. Transcription factor expression can be used to establish homology of different photoreceptor (PR) types across the insects: Prospero (Pros) for R7, Spalt (Sal) for R7+R8, and Defective proventriculus (Dve) for R1-6. Using gene knockout (CRISPR/Cas9) in houseflies, butterflies, and crickets and gene knockdown (RNAi) in beetles, we found that like *Drosophila*, EGFR and Sevenless (Sev) signaling pathways are required to recruit motion and color vision PRs, though *Drosophila* have a decreased reliance on Sev signaling relative to other insects. Despite morphological and physiological variation across species, retina development passes through a highly conserved phylotypic stage when the unit eyes (ommatidia) are first patterned. This patterning process likely represents an “insect eye ground plan” that is established by an ancient developmental program. We identify three types of developmental patterning modifications (ground plan modification, nonstochastic patterns, and specialized regions) that allow for the diversification of insect eyes. We suggest that developmental divergence after the ground plan is established is responsible for the exceptional diversity observed across insect visual systems.

## Significance

Insect eyes have adapted to a vast range of environments and natural histories, yet the compound eye remains fundamentally similar. We explore the genetic and developmental basis of this dichotomy. We identify a set of transcription factors that are expressed in homologous photoreceptor types across a wide range of species and find that distant groups use the same signaling pathways for photoreceptor recruitment. We find that flies are unique in using Sevenless signaling to recruit only the R7 photoreceptor. We identify three categories of modifications that adapt insect eyes to meet specific functional requirements. Deep conservation of patterning will simplify the search for the genetic basis of adaptation by making differences stand out.

The process of evolution has given rise to an astonishing array of animal forms, enabling species to adapt to diverse environments. While some structures and traits evolve rapidly across species, others remain relatively unchanged over long periods of evolutionary history. One such structure that has deeply conserved external morphology is the compound eye of insects and crustaceans. Compound eyes are composed of numerous individual repeating unit eyes (ommatidia), each with its own lens and photoreceptors (PRs). The precise morphology of this structure can vary widely across species, with differences in the number, size, and arrangement of ommatidia helping them to meet the functional requirements of complex behaviors such as foraging, navigation, detecting predators, and recognizing mates. These behaviors themselves are influenced by the natural history and ecology of each species. Despite this diversity, the underlying structure is still highly recognizable as a compound eye, begging the question of what genes are conserved in compound eye patterning and development, and which have been modified to generate diversity of form and function.

Retina development and PR recruitment has been heavily studied in *Drosophila melanogaster* [reviewed in (1, 2)]. Patterning and cell fate specification occur in the eye/antennal imaginal disc during the third larval instar. Eye patterning can be divided into three stages: specification of eye vs. noneye, initiation and progression of the morphogenetic furrow, and specification of cell types including the PRs, cone cells, pigment cells, and bristle cells. Genes that control initial eye specification such as *eyeless* (human PAX6) and *orthodenticle* (human OTX) have been found to be deeply conserved and establish the region that gives rise to the future retina (3–6). Next, the Hedgehog and Decapentaplegic signaling pathways coordinate progression of the “morphogenetic furrow” (7–9), a physical indentation which advances across the eye from posterior to anterior (1, 10). Morphogenetic furrow progression leaves behind evenly spaced clusters of cells called R8 equivalence groups that each refine into a single R8 PR using lateral inhibition (1, 11). R8 then uses the EGFR signaling and Spitz ligand to recruit PRs R2/5, which together again use EGFR/Spitz to recruit R3/4, then R1/6 (12–14). Finally, a combination of three signaling pathways is used to recruit the single R7 PR of each cluster: EGFR receives the ligand Spitz from R2/5, Notch receives the ligand Delta from R1/6, and the receptor tyrosine kinase (RTK) Sevenless receives the ligand Boss from R8 (15, 16). Together, each cluster of eight PRs subsequently recruit cone, pigment, and bristle cells and develop into an individual ommatidium. The uniform spacing of initial R8 specification leads to an evenly spaced crystalline array of ommatidia that make up the adult compound eye.

This dynamic recruitment process establishes PR identity and type, which is maintained by the expression of different transcription factors (TFs). PRs R7 and R8 are referred to as the “inner” PRs because of the central position of their light-gathering rhabdoms and they are primarily responsible for color vision (17–19). R1-6 “outer” PRs express broad-spectrum Rhodopsin 1 (Rh1) and are responsible for motion vision. Prospero (Pros) is a marker of R7 PR fate and is critical for R7 specification (20). In *Drosophila*, Spalt (Sal) is transiently expressed in R3/4 before turning on in R7 and R8, where expression is maintained through pupal and adult stages (21, 22), where it is required for expression of color-sensitive Rh3, Rh4, Rh5, and Rh6 in inner PRs (21). Defective proventriculus (Dve) is expressed at high levels in R1-6 where it represses color-sensitive Rh3/5/6 and promotes expression of Rh1 (23). After this initial patterning, a stochastic patterning process establishes two ommatidial subtypes via the decision to express (or not express) a single TF Spineless (Ss) (24). Dve is expressed downstream of Ss at low levels in the Ss-ON subset of R7s that will later express Rh4, making Dve not exclusive as an outer PR marker (2, 25). This Dve expression in R7 occurs at low levels, as opposed to high Dve in outer PRs.

Outside *Drosophila*, studies that examined the morphology of developing and adult insect retinas have suggested that similar mechanisms might pattern the retinas of a wide range of species (26–29). The morphology of early-stage retina patterning has been characterized in only a few well-established systems, including *Tribolium* (red flour beetles) (30, 31), *Ephestia* (flour moths) (32), *Gryllus* (crickets) (33), and *Schistocerca* (grasshoppers) (34). Each of these species exhibits a superficially similar retina patterning process, with the regular structure of the compound eye appearing from posterior to anterior and through sequential addition of differentiated cells into each developing ommatidium, similar to what has been observed in *Drosophila*. Adult retina morphology has been characterized in many additional groups, but assessing PR homology across species based on PR position and axonal projection pattern via electron microscopy (EM) can be painstaking and impractical to replicate in every species, and this approach has not always produced a reliable assessment of homology (35, 56). Recently, new tools have made it possible to begin to characterize gene expression and gene function even in nonmodel species such as beetles, butterflies, and bees (36, 37). For example, the TF Glass has been found to be essential for PR differentiation and this role is conserved between *Drosophila* and *Tribolium* (38, 39). Nonetheless, whether the same genes and signaling pathways are used in PR recruitment across the insects remains largely unexplored.

Here, we first set out to test whether TFs that are known to be required for the specification of different PR types in *Drosophila* are expressed in similar patterns across diverse insect species. Next, we examined conservation of the role of Sevenless/Boss (RTK) and EGFR/Spitz signaling using CRISPR/Cas9 or RNAi in multiple insect species. Our comparative analysis of TFs and signaling pathways revealed a high degree of similarity in the developmental patterning of compound eyes even among distantly related insect species, suggesting that a common ground plan is used in insect eye development. Finally, we identify three categories of modifications to eye patterning across groups: 1) Ground plan modification: In rare cases, the ground plan itself can be modified, as in the addition of a second R7 PR in the Lepidoptera, thought to have allowed for expanded color vision; 2) The evolution of nonstochastic patterns: Rh expression patterns can be reorganized into nonstochastic patterns. This has happened in groups such as Dolichopodid flies, mosquitos, and crickets; 3) The evolution of specialized regions. This includes dorsal polarized light detectors and specialized regions of the eye used in target detection, which are often sex-specific. We discuss examples of visual system modifications that fit into these categories and provide data for each.

## Results

### A Core Set of TFs Pattern the PRs of Diverse Insect Species.

**Fig. 1.**
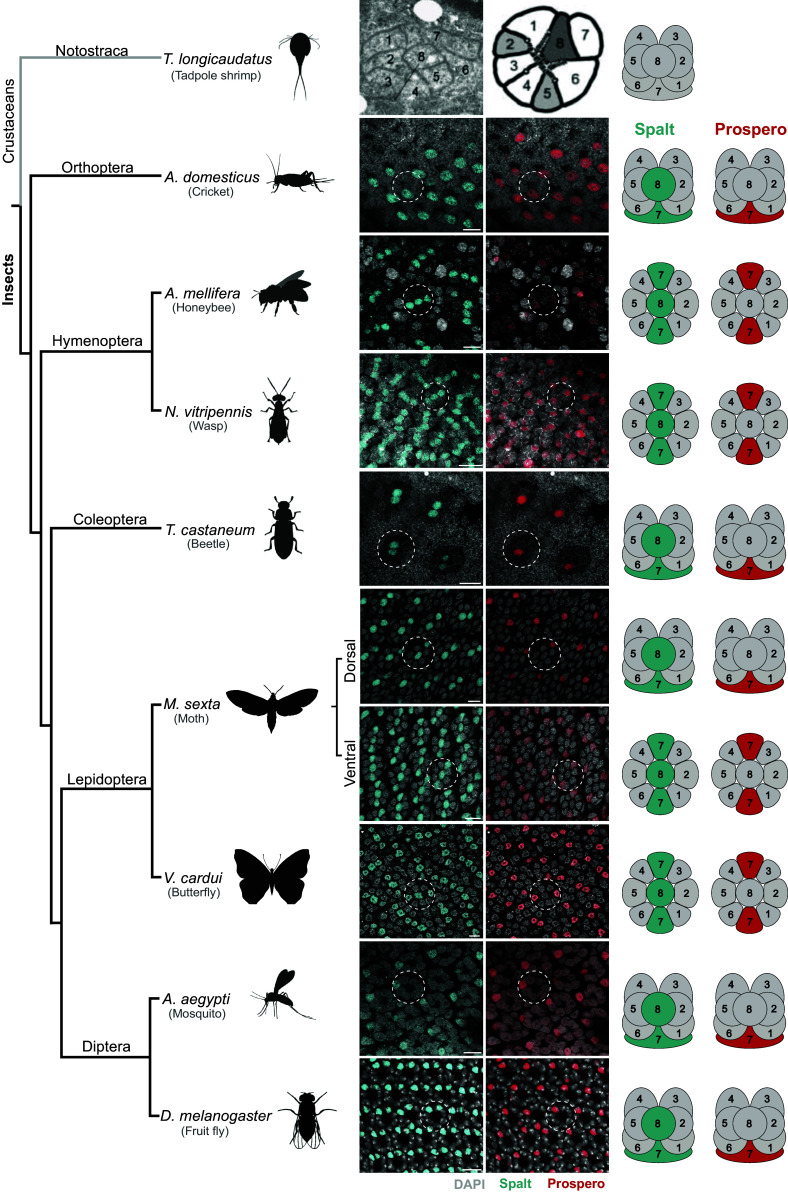
The TFs Spalt and Prospero are expressed in a subset of PRs in developing retinas of species across the insects and can be used to assign PR homology. Phylogenetic relationships between insect groups [as in (49)] are shown on the *Left*. In immunohistochemical stains of developing retinas, Spalt (cyan) labels R7 and R8 PRs while Prospero (red) labels R7 PRs. DAPI (gray) labels all nuclei. White circles outline a single representative ommatidium in each species. Schematics of single ommatidia are shown on the *Right*. *Acheta domesticus* retinas were examined at embryonic stages, *A. mellifera*, *N. vitripennis*, *T. castaneum*, *V. cardui*, *M. sexta*, *A.aegyptic*, and *D. melanogaster* tissues were collected during the pupal stage. *T. longicaudatus* represents an outgroup to the insects and in a previous study using EM was shown to have eight PRs. Insect schematics created with BioRender.com. Images of *T. longicaudatus* used with permission from (26). (Scale bars, 10 µm.)

**Fig. 2.**
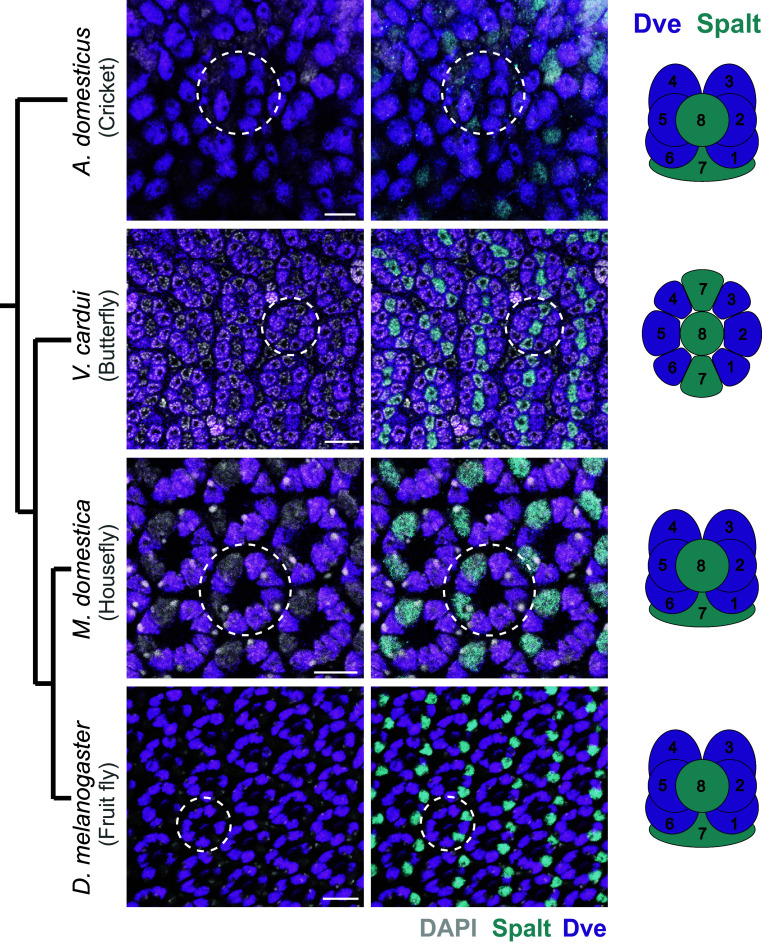
The TF Dve is expressed in a subset of PRs in developing retinas of selected species and labels outer PRs, mutually exclusive with the Sal-expressing inner PRs. Dve (magenta) and Sal (cyan) expression is shown in *A. domesticus*, *V. cardui*, *M. domestica,* and *D. melanogaster*. DAPI (gray) labels all nuclei. White circles outline a single representative ommatidium in each species. (Scale bars, 10 µm.)

We used cross-reactive antibodies to the TFs Sal, Pros, and Dve to examine their expression in a diverse set of species across the insects (Figs. 1 and 2). In *Drosophila*, this combination of TFs labels the inner PRs R7 and R8 (marked by Sal), R7 (Pros+Sal), and R8 (Sal alone) (Fig. 1, *Bottom*). Due to differences in when the eye is patterned in each group, we first identified stages where we could observe PR recruitment and where ommatidia are readily comparable using these markers.

Similar to *Drosophila*, ommatidia in the yellow fever mosquito *Aedes aegypti* (Diptera), the red flour beetle *Tribolium castaneum* (Coleoptera), and the house cricket *A. domesticus* (Orthoptera) contain eight PRs, with two PRs marked by Sal and a single Sal-positive PR also marked by Pros (Fig. 1). The six Sal-negative PRs are marked by Dve expression in *Drosophila*, house flies *Musca domestica* (Diptera), house crickets (*A*. *domesticus*) (Fig. 2), and mosquitos *Aedes* and *Anopheles* (23) suggesting they each have six outer PRs. This assessment matches the identity of these cells as previously assigned by their relative positions in adult retinas. In cricket, PRs previously shown to have short visual fiber axonal projections express Dve, while long visual fiber PRs are in positions that we find to express Sal (40). We conclude that molecular markers support this assessment of homology, based previously on PR position and axonal projection pattern, with eight PRs total, six outer PRs, and two inner PRs per ommatidium even in a distantly related outgroup species, the cricket *A. domesticus*.

In contrast, ommatidia in the honeybee *Apis mellifera* (Hymenoptera), the parasitoid wasp *Nasonia vitripennis* (Hymenoptera), and the butterfly *Vanessa cardui* (Lepidoptera) have nine PRs and two are Pros-positive (Fig. 1). We showed previously that butterflies have two R7-type PRs (36). In the two hymenopteran species examined, marker TF expression suggests that the additional PR is also an R7-type PR. This was first proposed for *Apis* based on morphology (29), and molecular markers now support this hypothesis (Fig. 1). Interestingly, in the moth *Manduca sexta* (Lepidoptera), we observed that ommatidia in the dorsal region are fly-like and have eight PRs with one R7, while ommatidia in the ventral region are butterfly-like and have nine PRs with two R7s. A previous study of morphology noticed this difference in PR number in *M. sexta* but did not speculate as to which PR types were present in dorsal vs ventral regions (41). Molecular markers indicate that this difference in PR number is due to a differing number of R7s.

Overall, PR number and arrangement during early developmental stages is strikingly conserved across the species examined. We show that these markers allow for assessment of PR number and homology even in species where a detailed evaluation of ultrastructure and PR connectivity has not been performed, such as in *Nasonia* wasps and *Acheta* crickets. Together, these results demonstrate that molecular marker expression can be used to establish homology of PR types and that patterning is deeply conserved.

### TF Expression can be Used to Examine the Order of PR Recruitment in Diverse Species.

**Fig. 3.**
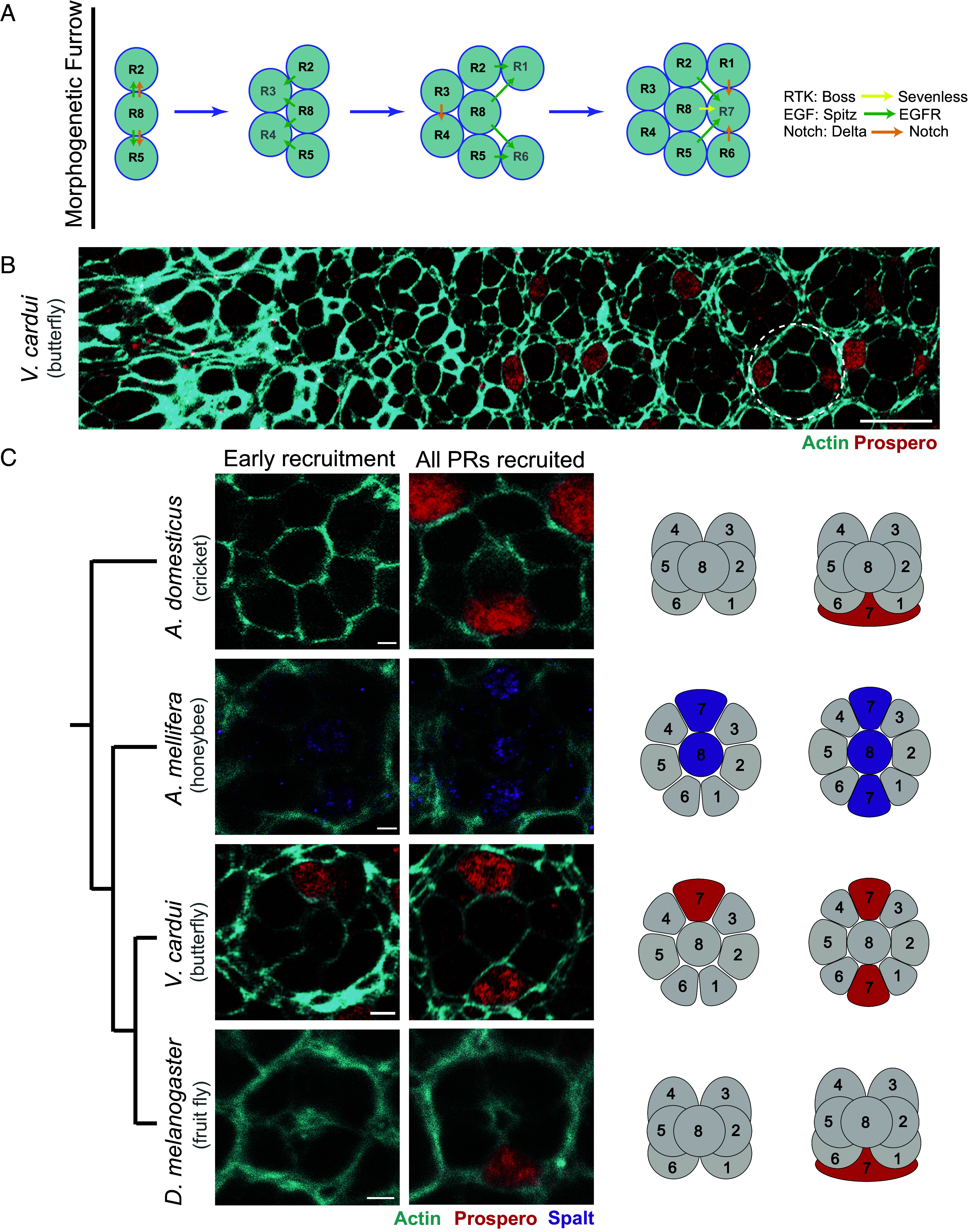
Molecular markers can be used to assess the order of PR recruitment in various species. (*A*) Schematics show the order of PR recruitment in *D. melanogaster* in the eye imaginal disc during the third larval instar, as well as the signaling pathways used at each step. Adapted from (43). (*B*) Confocal image of an early pupal *V. cardui* retina, showing a region from the morphogenetic furrow on the left to differentiated ommatidia on the right. Anterior is to the left. PR cells are outlined by actin/phalloidin staining (cyan), and R7 PRs are labeled by Pros (red). (Scale bar, 10 µm.) (*C*) Images at two stages of PR recruitment are shown for four species. Phylogenetic relationships are shown to the *Left*. Ommatidia are stained for actin/phalloidin (cyan) showing cell outlines, and either Sal (magenta) or Pros (red). The first PR recruited becomes Sal-positive and Pros-negative, indicating that it is homologous to *Drosophila* R8. In *A. mellifera* and *V. cardui* one R7 PR is recruited earlier than the other and appears in the position of the mystery cell in *Drosophila*, across from R1/6 and opposite the later-recruited R7. (Scale bars, 2 µm.)

We next asked whether PRs are recruited in a similar order during development. Fig. 3*A* shows this order of PR recruitment in *Drosophila* and summarizes the signaling pathways involved during each step (16, 42). We used immunohistochemistry to examine PR specification over time in the cricket *A. domesticus*, the honeybee *A. mellifera*, the butterfly *V. cardui*, and the fruit fly *D. melanogaster*. Images in (Fig. 3 *B* and *C*) show the outlines of newly formed ommatidia during the process of PR specification. PR recruitment occurs sequentially over time, with Sal or Pros turning on shortly after specification of R8 or R7 fate. A view of this process across the anterior/posterior is shown for the butterfly *V. cardui* in (Fig. 3*B*). In (Fig. 3*C*), we show comparable time points for each species. This process is similar even between distantly related *Drosophila* fruit flies and *Acheta* crickets. The last PR recruited in each species expresses both Sal and Pros, identifying it as an R7 PR (Fig. 3). The selected Hymenoptera and Lepidoptera species sequentially recruit two R7 cells, with one recruited earlier than the other. It has been hypothesized that this additional R7 is recruited in the position of the “mystery cell” of *Drosophila*, between R3 and R4 (36), which is consistent with the order of recruitment, position, and the relative timing of appearance that we observed in both butterfly and honeybee. Together, these results suggest that the order of recruitment and relative positions of where different PR types are recruited is highly conserved.

### Sev Is Broadly Used for the Recruitment of R7 as Well as Four Outer PRs.

**Fig. 4.**
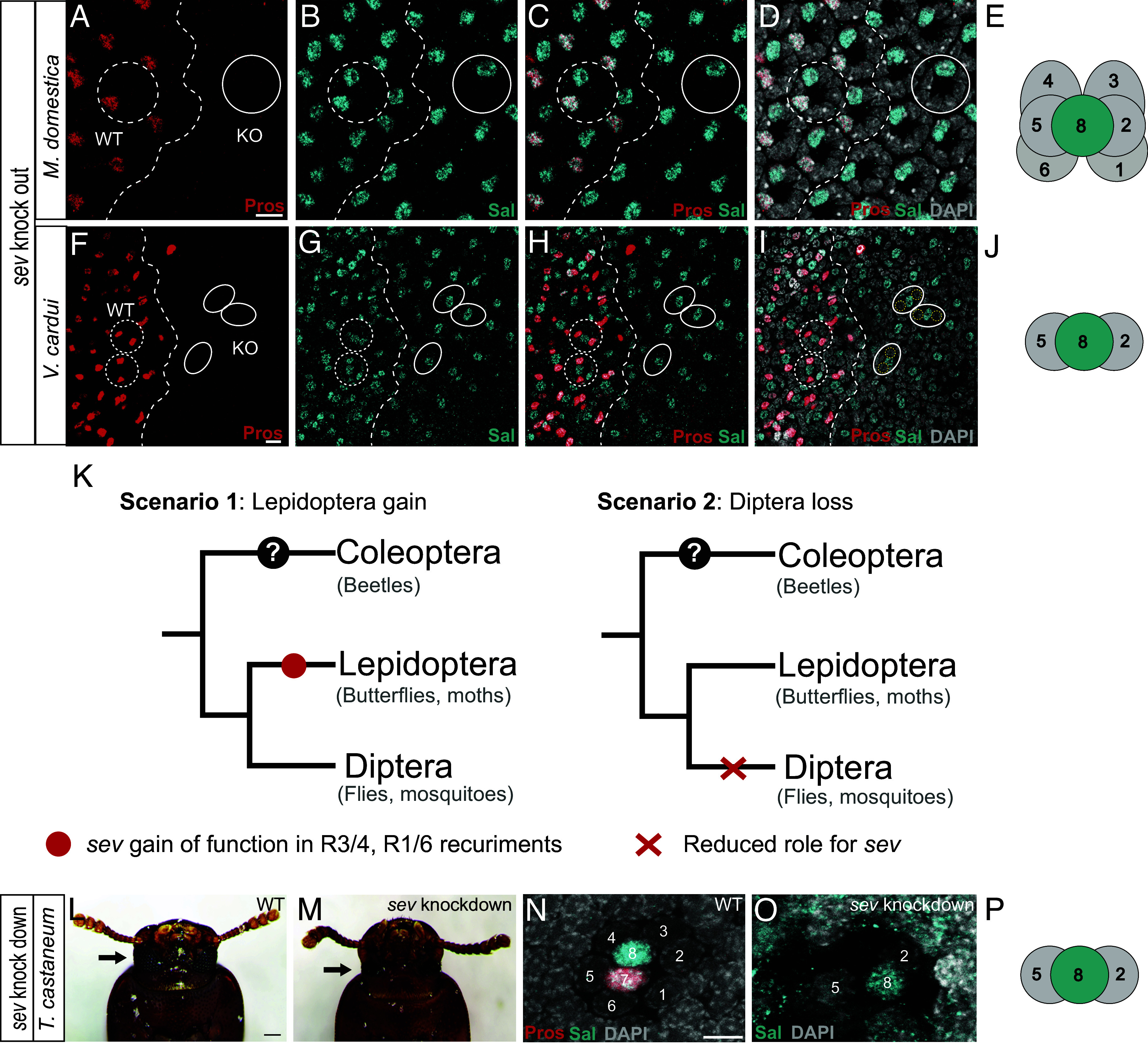
Sev (RTK) signaling plays a larger role in PR recruitment in insects outside the Diptera. (*A*–*E*) CRISPR-induced mosaic knockout of *sev* results in the loss of R7 cells in pupal retinas of the house fly *M. domestica*. R7s stained with Pros (red) in WT regions are lost in *sev* KO regions (compare WT to KO regions in *A*). Similarly, in WT regions Sal staining (cyan) is observed in R7 and R8 but only the R8 remains in *sev* KO regions, labeled by Sal-expressing (cyan) but not Sal+Pros coexpressing (white) nuclei (compare WT to KO regions in *B–D*). In (*D*), all nuclei are labeled with DAPI (gray). The boundary between WT and *sev* KO regions is indicated by a white dashed line. A dashed white circle highlights an example ommatidium in a WT region, while a solid white circle highlights an example ommatidium in a *sev* KO region. WT, wild type; KO, knockout. (Scale bars, 10 µm.) (*E*) Schematic shows the loss of R7 in *M. domestica*. (*F–J*) In *V. cardui* butterflies, CRISPR-induced mosaic knockout of *sev* results in loss of two R7s and four outer PRs per ommatidium. In WT pupal retina regions, two R7s per ommatidium express Pros (red), and loss of Pros signal in regions disrupted by *sev* KO indicates loss of both R7s [compare WT to KO regions in (*F*)]. Similarly, Pros + Sal coexpressing R7 cells are lost but not Sal-only expressing cells (R8s) [compare WT to KO regions in (*G–I*)]. Unexpectedly, additional outer PR nuclei are missing in *sev* KO regions, based on the number of nuclei remaining and that only two of these are Dve positive (SI Appendix, Fig. S1). The boundary between WT and *sev* KO regions is indicated by a white dashed line. Dashed circles show examples of ommatidia in WT region, and solid circles highlight examples of ommatidia in *sev* KO region, where Sal-positive R8 remains, and only two neighboring PR nuclei (yellow dashed circles) remain. All nuclei in (*I*) are labeled with DAPI (gray). WT, wild type; KO, knockout. (Scale bars, 10 µm.) (*J*) Schematic shows the loss of R7s and outer PRs R1/6, R3/4 in *V. cardui*. (*K*) Two scenarios for how the role of Sev might have evolved. In Scenario 1, Sev gained expanded function in recruitment of four outer PRs in the butterfly lineage, while in Scenario 2, Diptera evolved a decreased reliance on Sev signaling and no longer require Sev for outer PR recruitment. (*L–O*) RNAi knockdown of Sev in *T. castaneum* results in loss of R7 and four outer PRs. (*L* and *M*) Adult eyes (arrows) in wild-type (*L*) vs. Sev knockdown (*M*) are severely reduced in size. (Scale bars, 160 µm.) (*N* and *O*) Comparing WT (*N*) to Sev knockdown pupal retinas (*O*) shows that Pros-positive R7 (red) is lost, Sal-positive/Pros-negative R8 remains, and only two neighboring PR nuclei remain. Based on order of recruitment and that R2/5 are required for recruitment of the next four outer PRs, the remaining two are most likely R2/5. (Scale bars, 10 µm.) (*P*) Schematic shows the loss of R7 and outer PR R1/6, R3/4 in *T. castaneum*. This result supports Scenario 2 and suggests that the ancestral insect used Sev signaling in the recruitment of R3/4/1/6 as well as R7.

We next asked whether the same signaling pathways used in *Drosophila* are used in PR recruitment in other species for recruitment of R8, R1-6, and for one or two R7s. *Drosophila* RTK “Sevenless” mutants lack R7 PRs and this mutation was used in genetic screens to identify important components of the RTK signaling pathway (44, 45). We examined conservation of Sev signaling in R7 PR recruitment in *Musca* houseflies and *Vanessa* butterflies using CRISPR/Cas9 gene knock out. As in *Drosophila*, loss of *sev* in *Musca* resulted in the loss of R7 PRs, while other PRs were unaffected (Fig. 4 *A–E* and SI Appendix, Fig. S1 *A–C*). In contrast, when we disrupted *sev* in *Vanessa* butterflies, we found that the absence of R7 PRs was accompanied by the loss of four outer PRs as well as the loss of a variable number of cone cells (Fig. 4 *F–J* and SI Appendix, Fig. S1*F*). Examples of characteristic ommatidia that have just two Dve-positive outer PRs remaining is shown in SI Appendix, Fig. S1*E* (yellow circles). Other remaining nuclei in ommatidia in these regions are Dve-negative and are likely pigment or bristle cell nuclei. Because R2/5 are recruited first and required for the recruitment of R3/4 and R1/6, the remaining two Dve-positive PRs are likely R2/5. By examining the edges of *sev* knockout clones, we observed examples of ommatidia where only the R7 was missing (SI Appendix, Fig. S1 *D* and E), supporting the idea that Sev is used directly for R7 recruitment, as well as in the recruitment of R3/4/1/6. In these experiments, unlike in *Drosophila* where loss of Sev has no known phenotype other than R7 loss (44), CRISPR disruption of Sev in *Vanessa* was highly lethal with only a small number of “mosaic mutant escapers” reaching pupal stages, suggesting that Sev also plays a more significant role in other tissues at earlier developmental stages in butterflies.

The loss of outer PRs upon Sev KO in butterflies (in addition to R7 loss) suggests that butterflies either 1) have an increased reliance on Sev signaling during PR recruitment compared to other insects (Fig. 4*K*, “Scenario 1”), or 2) that flies have a reduced role for Sev in PR recruitment compared to the ancestral role, using it only for R7 specification (Fig. 4*K*, “Scenario 2”). To distinguish between these hypotheses, we examined Sev function in an outgroup, *Tribolium* red flour beetles (Coleoptera). We used RNAi knockdown and found that loss of Sev results in the loss of R7 and four outer PRs, leaving only the central Sal-positive R8 PR and two flanking outer PRs (compare Fig. 4 *N* and *O*) and severely disrupted eye morphology (Fig. 4 *L* and *M*). The loss of four outer PRs as well as the R7 PR in each ommatidium matches the phenotype observed in butterfly Sev CRISPR mutants, supporting Scenario 2 and indicating that *Drosophila* have an evolutionarily reduced role for Sev signaling in PR recruitment.

### The Use of EGFR/Spitz in PR Recruitment Is Highly Conserved.

We next investigated the role of the EGFR/Spitz signaling in PR specification in the butterfly *V. cardui* and the cricket *A. domesticus*. In *Drosophila*, the EGF receptor is used together with the ligand Spitz in the sequential rounds of PR recruitment shown in Fig. 3*A* (green arrows). It is required for recruitment of all six outer PRs R1-6 as well as R7 (46–48). The EGF receptor plays other roles such as the establishment of R8 spacing using other EGF ligands such as Keren (48), but loss of Spitz does not disrupt initial ommatidial spacing or specification of R8 PRs. Loss of Spitz severely disrupts patterning shortly after R8s are specified in a regular grid, and this is soon followed by widespread cell death in those regions (46).

**Fig. 5.**
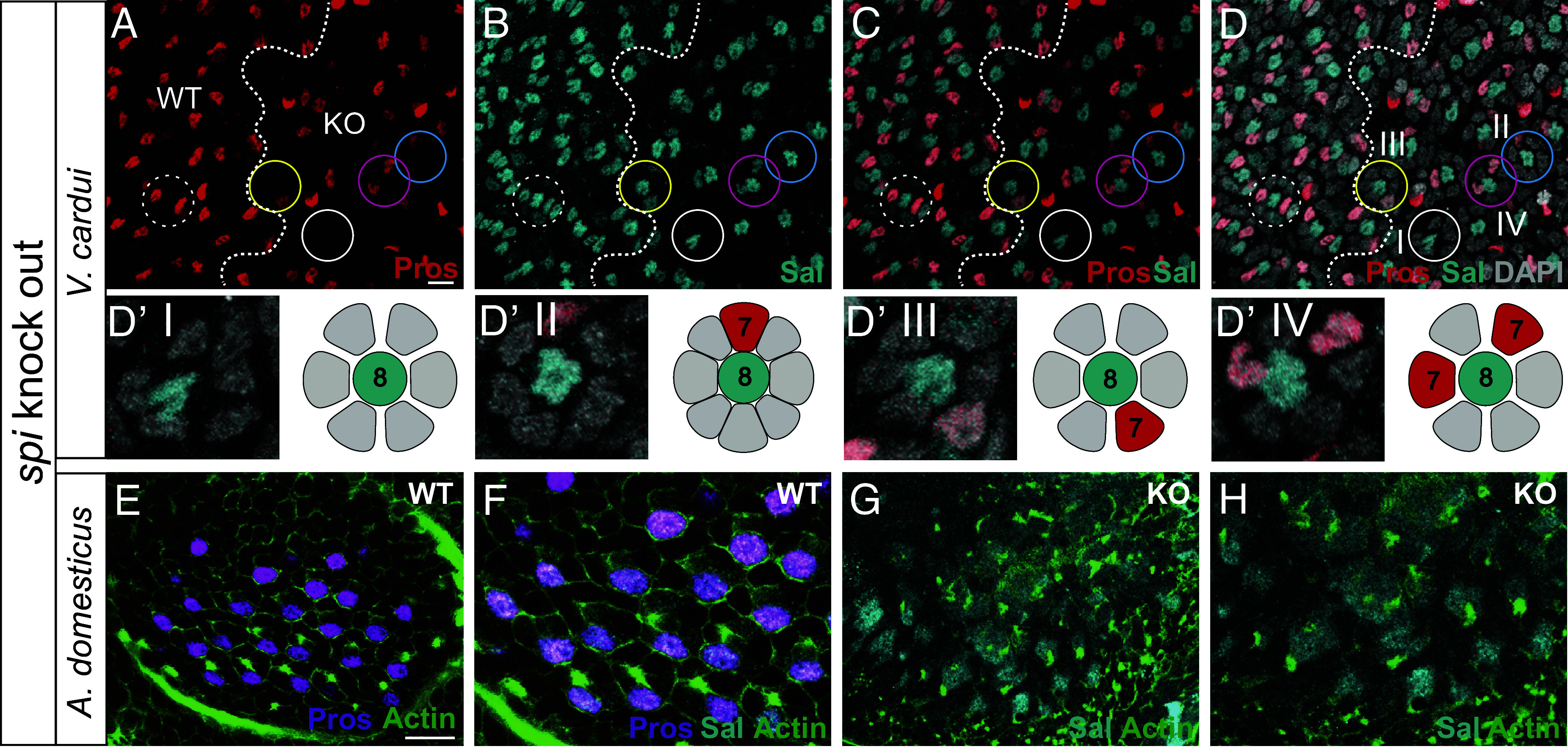
EGFR/Spitz plays a conserved role in PR recruitment. (*A*–*D*) CRISPR mosaic knockouts of *spitz* result in the loss of R7 and outer PRs in pupal retinas of *V. cardui*. Dashed circles show example ommatidia in WT region; compare to solid circles in *spitz* KO regions. The boundary between WT and disrupted *sev* KO regions is indicated by a white dashed line. In the WT region, Pros (red) is expressed in two R7s while *sal* (cyan) is expressed in R8 and two R7s per ommatidium. In KO regions, we observed a range of phenotypes, suggesting that a mix of null and hypomorphic mutations are present. In *Drosophila*, null mutant regions rapidly undergo cell death (48), and so a mix of clone types is useful in this case. Some ommatidia lack Pros (red) signal, indicating a loss of both R7s (white circle; *D’I*). Other ommatidia lack one R7 and have variable numbers of outer PRs (yellow or cyan circle, *D’II* and *III*), while yet other examples have two R7s but missing outer PRs (magenta circle; *D’IV*). Sal (cyan) is expressed in R8 (which also lack Pros) in both WT and KO region. (Scale bars, 10 µm.) (*E* and *F*) In WT embryonic retinas of *A. domesticus*, Pros (magenta) is expressed in R7, Sal (cyan) is expressed in both R7 and R8. (*G* and *H*) *A. domesticus* CRISPR mosaic retinas mutant for *spitz* are severely disrupted and are comparable to *Drosophila spitz* mutant clone regions, with only R8s specified in a regular grid (single, evenly spaced Sal-positive nuclei shown in cyan) and likely loss of all other PRs. (Scale bars, 10 µm.). Pros (magenta) staining is lost and the regular grid, as visualized by actin/phalloidin staining (green), is disrupted. We conclude that Spitz is necessary for proper PR recruitment.

In *Vanessa* butterflies, CRISPR disruption of Spitz caused significant early lethality, but we were able to obtain mosaic mutant escapers that exhibit disrupted patterning in portions of the retina (Fig. 5 *A–D*). In making mosaic somatic mutant animals it is not uncommon to produce a mixture of null and hypomorphic clones where the protein is partially functional. We observed regions with ommatidia that contained variable numbers of PRs (Fig. 5 *A–D*) including ommatidia with one or both R7s missing, ommatidia where the R7 appears to be transformed into an outer PR, and examples where some outer PRs are missing (Fig. 5 *D*’*I*–*IV*). While hypomorphic clones can produce variable phenotypes, the loss of both outer PRs and R7s, as well as the conversion of R7 to outer fate, suggests that Spitz is required for the specification of both R7 and outer PRs.

In *Drosophila* Spitz loss-of-function experiments, regions of cells containing a null mutation allow only R8 PRs to be specified (46). In *Acheta* crickets, KO of *spi* resulted in severely disrupted retina patterning, and we observed evenly spaced Sal-positive nuclei with no neighboring Pros or Dve-positive nuclei, suggesting that only R8 PRs are specified (Fig. 5 *E*–*H*), similar to the outcome in *Drosophila*. We conclude that EGFR/Spitz signaling plays a highly conserved role in PR recruitment in distantly related insect species.

### Modifications Used in the Diversification of Insect Eyes.

**Fig. 6.**
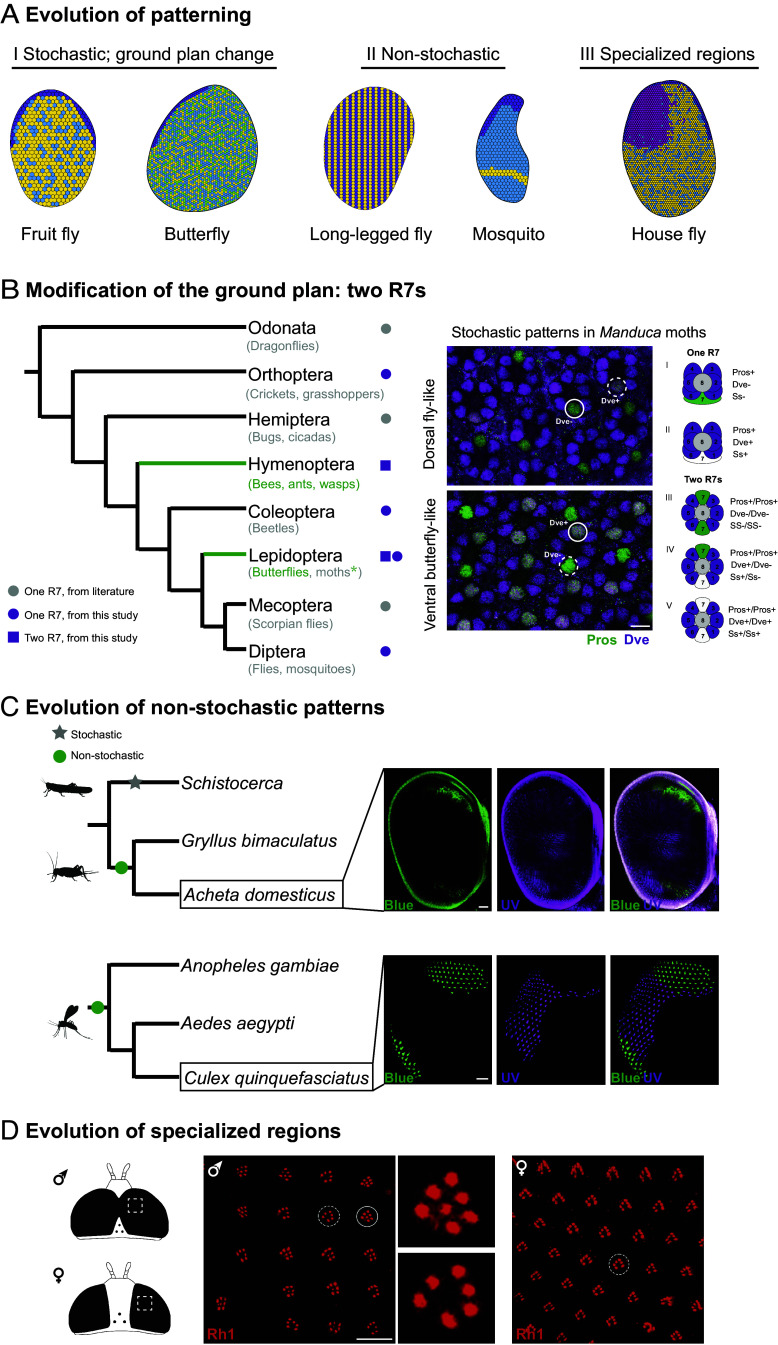
Three types of modifications to the insect eye ground plan. (*A*) Retina schematics summarize differences in patterning and three categories of modifications that are used to modify insect visual function. (*B*) Modification of the insect eye ground plan itself: Lepidoptera and Hymenoptera have ommatidia that contain two R7 PRs. A phylogeny of insect groups is shown on the left, with relationships taken from (49). Gray circles indicate evaluation via morphology in previous studies, while purple circles and squares indicate that additional data are presented in this study. On the right, antibody stains in the hawkmoth *M. sexta* show fly-like ommatidia in the dorsal region (eight PRs, one R7) and butterfly-like ommatidia in the ventral region (nine PRs, two R7s). Dve expression (magenta) in Pros-positive (green) R7s can be used as a marker of ommatidial subtype and stochastic patterning, and shows that the additional R7 is correlated with the production of three stochastically distributed ommatidial types in the ventral retina. (Scale bars, 10 µm.) (*C*) In a second type of modification, some groups have lost stochastic patterning. *S. gregaria* grasshoppers were previously shown to have stochastically patterned retinas (34), while *G. bimaculatus* crickets were found to have regionalized patterns (50). We examined Rh expression as a marker of ommatidial type in an additional cricket species *A. domesticus* and find that it has regionalized, nonstochastic expression similar to *Gryllus*, with the blue-sensitive Rh localized to the ventral retina (as well as the dorsal rim area). (Scale bar, 100 µm.) In a similar but likely independent example of the evolution of nonstochastic patterns, *Aedes* and *Anopheles* mosquitos were found to have regionalized Rh expression (51). We examined Rh expression in *C. quinquefasciatus* mosquitos and find a similar nonstochastic pattern, with a narrow ventral stripe of blue-sensitive Rh expression (as well as in a large dorsal rim area), suggesting that stochastic patterning was lost early in mosquito evolution. (Scale bar, 50 µm.) (*D*) A third type of modification interrupts stochastic patterns, such as the male-specific “Love Spot” region in *Musca* house flies and other Dipterans. We show male-specific expression of Rh1 in dorsal R7 PRs in the hover fly *Sphaerophoria sp*., suggesting that they have similarly modified R7 PRs to the previously characterized feature in *Musca*. (Scale bar, 20 µm). Data sources in (*A*): Mecoptera from (53), Hemiptera from (54), Odonata from (55).

We present evidence that insect eyes are patterned using deeply conserved patterning genes and signaling pathways and have highly similar underlying structure (Figs. 1–5). Yet insects live in a vast array of environments and have varied visual requirements. What types of changes to developmental patterning help adapt insects to diverse environments and natural histories? We provide examples of three main categories of patterning changes that modify insect eyes for specific function (overview in Fig. 6*A*).

#### Type 1 evolution of changes to the ground plan.

Examples of developing retinas shown in Fig. 1 and extensive literature on insect eye morphology indicate deep conservation of an eight-PR arrangement across the insects [reviewed in (56)], with two main exceptions. Examples in both the Lepidoptera and Hymenoptera have ommatidia that contain nine PRs instead of eight (Figs. 1, 2, and 6*B*). Outgroups to both orders, such as the Orthoptera (crickets and grasshoppers), have eight PRs per ommatidium. Whether ommatidia with two R7s evolved once and were lost multiple times or evolved twice, once in each group, is an open question (Fig. 6*B*). The loss hypothesis would require it to have been lost in groups that diverged between the Hymenoptera and Lepidoptera (such as in the beetles, Coleoptera) and again in the lineage leading to the Diptera. Alternately, it could have been gained once in the lineage leading to the Hymenoptera and again in the Lepidoptera.

For the loss hypothesis, modern molecular phylogenies (e.g., ref. 49) suggest that several orders of insects arose between the Hymenoptera and the Lepidoptera, which would have required the loss of two R7s in at least the Coleoptera, Megaloptera, and Neuroptera. Morphological data show that species in these orders have eight PRs (31, 32, 57). In Fig. 1 we show evidence that indicates that one of these groups, *Tribolium* beetles, has a single Pros-positive R7. In this scenario, an additional loss would have occurred in the lineage leading to the Diptera, suggesting it would have required at least four independent losses in total.

Alternately, it is possible that the gain of a second R7 PR per ommatidium occurred independently in the two groups. A previous review using data on adult PR morphology concluded that the ancestral state was most likely ommatidia with eight PRs, and that the Hymenoptera and Lepidoptera independently evolved additional PRs (56). Our survey of molecular marker expression supports this view. To test this hypothesis further, we examined *M. sexta* hawkmoths, which had previously been shown to have eight PR in the dorsal retina and nine PR in the ventral retina (41). In antibody stains, dorsal *Manduca* retinas are fly-like with six Dve-positive outer PRs and a single Pros-positive R7 (Fig. 6 *B* *Right*, *Top*). Ventral *Manduca* retinas are butterfly-like and contain nine PRs, with six Dve-positive outer PRs and two Pros-positive R7 PRs (Fig. 6 *B* *Right*, *Bottom*). The presence of both fly-like and butterfly-like ommatidial types in the retinas of a single moth species provides additional evidence that this trait may have evolved within the Lepidoptera. Together with phylogenetic placement, and because two gains is more parsimonious than four or more losses, we conclude that ommatidia containing two R7s most likely evolved independently in the Hymenoptera and Lepidoptera.

The evolution of a second R7 PR per ommatidium produced more complex retinal mosaics and allowed for expanded color vision in butterflies (36). In *Drosophila*, a stochastic choice of whether to express the TF Ss is made in individual R7s and controls the decision of what Rh to express (24), as well as a coordinated R8 PR Rh choice via activin signaling (58). This Ss-ON/Ss-OFF decision is cell intrinsic and controls the ratio of ommatidial types produced (59). In butterflies, the presence of a second R7 allows for Ss-ON/ON, ON/OFF, and OFF/OFF combinations in the two R7 cells, providing additional information that is required for the production of three stochastically distributed ommatidial types [SI Appendix, Fig. S3; (36)]. Over evolutionary time, new Rhs have come to be expressed in different combinations of PRs that rely on this additional identity, such as a red-sensitive Rh that provides the ability to see red colors in swallowtail butterflies (Papilionidae) in one of the three ommatidial types that result from having two R7s (36, 60). We tested whether *Manduca* use similar genes as butterflies to make additional stochastic choices in the portion of their retinas that have two R7 PRs. Using Dve expression in the Pros-positive R7s as a readout of Ss-ON/Ss-OFF status (36), we observed patterns that match previously published Rh expression data (Fig. 6 *B* *Right*). Together, these data suggest that the dorsal *Manduca* retina has two stochastically distributed ommatidial types while the ventral retina has three.

#### Type 2 evolution of nonstochastic patterning.

After the initial ground plan is established, the retina receives additional patterning. In *Drosophila*, the stochastic ON/OFF decision to express Ss controls ommatidial subtype and which Rhs are expressed, and therefore which wavelengths are detected. This secondary patterning system using Ss might be expressed in different patterns in the retinas of some species. For example, while Rh or patterning gene expression has not yet been characterized in the Dolichopodidae (the long-legged flies), corneal lens color and underlying morphology has become nonstochastic in some species (schematic in Fig. 6*A*) (61, 62). In a different spatial arrangement, *Aedes* and *Anopheles* mosquitos have lost stochastic patterning and instead produce a regionalized ventral stripe of Rh expression (schematic in Fig. 6*A*) (51). Similarly, while *Schistocerca gregaria* grasshopper patterning is stochastic (63), Rh patterning in *G. bimaculatus* crickets has been shown to be mosquito-like and regionalized (50). That specific groups have become regionalized hints at potential function, and it has been suggested that some species might use the ventral region as a ventral polarized light detector (35). To further examine the origins of regionalized patterning in crickets and mosquitos, we examined Rh expression in additional species.

We used HCR in situ hybridization to visualize expression of UV and blue-sensitive Rhs in the retinas of house crickets *A. domestica* and in mosquitos *Culex quinquefasciatus*. While the main region of the eye expresses UV-sensitive Rh in R7 PRs, we observed a regionalized patch of blue-sensitive Rh expression in ventral *A. domestica* retinas, similar to the pattern previously observed for *G. bimaculatus* (Fig. 6*C*). Though *Gryllus* and *Acheta* are closely related, this result shows that regionalized patterns are found across multiple genera and are not restricted to *Gryllus*. Further work will be needed to determine where regionalized patterns originated within the Orthoptera. In mosquitos, *Ae. aegypti* produces a narrow ventral stripe of blue-sensitive Rh expression while *An. gambiae* have a similar but broader ventral region (51). To improve phylogenetic coverage within the Culicinae, we examined a third laboratory species, *C. quinquefasciatus,* and find that they have a similar ventral region of blue-sensitive Rh expression in R7 PRs (Fig. 6*C*). The finding that all three species have regionalized patterns suggests that the origin of regionalized retinas occurred early, before the diversification of mosquitos. The evolution of nonstochastic patterns is one way in which insect retinas have been modified for specialized function.

#### Type 3 evolution of specialized regions.

We classify another type of retina modification as “specialized regions”, which we define as modified subregions of the retina that interrupt normal stochastic patterning. One example is the “dorsal rim area” (DRA), a region at the top of the retina used to detect the direction of polarization of skylight and used for navigation (Fig. 6*A*, dark purple). This region varies in shape and position in different species (35). In another example, a number of species in families across the Diptera have a male-specific region of the eye. This region interrupts normal stochastic patterning in males and is thought to provide increased sensitivity for target detection and tracking, and to aid in aerial pursuit of females that leads to mating [reviewed in (52)]. It has been colloquially called the Love Spot (64).

Similar male-specific regions are found in *Lycaena* copper butterflies (65), *Apis* honeybees (66), and in “turbinate” mayflies (67). Another interesting example of a sex-specific target detector, but this time in females, is found in the “big-headed flies”, in the Pipunculidae. Females in the genus *Chalarus* have enlarged Love Spot-like ommatidia in the frontal eye, likely used in the detection of moving oviposition sites: hopping tree- and leaf-hopper nymphs on the underside of leaves (68, 69). While external morphology of such features has been examined in many species, the most detailed work has been performed using house flies *M. domestica*, where electrophysiology, ultrastructure, and axonal connectivity have been closely examined (52, 64). Previous results from electrophysiological recordings suggest that they use broad spectrum-sensitive Rh1 in their outer PRs for motion vision (like *Drosophila*) and also in Love Spot R7 PRs. Rh expression has not been examined in other fly species with similar features. We used immunohistochemistry to examine Rh1 expression in a phylogenetically distant species of Syrphidae hover fly, *Sphaerophoria sp*., where we collected a mating male/female pair. While in the female retina Rh1 expression was limited to outer PRs, in males, we observed Rh1 expression in a subset of R7 PRs in the dorso-frontal region of the eye (Fig. 6*D*), providing an example of a specialized region with features similar to those characterized in *Musca*.

## Discussion

In this work, we selected a set of TFs and signaling pathways that are known to be important for PR recruitment in *Drosophila* and evaluated their role across the insects. We found that the TFs Pros, Sal, and Dve serve as reliable molecular markers and can be used to establish PR homology, and that their expression can be used to examine the order of PR recruitment. We establish that two of the same signaling pathways are used in similar ways to recruit PRs across insect groups, but with a shift in reliance from Sevenless (RTK) to EGFR/Spitz for the recruitment of four outer PRs in the Diptera. There is no apparent functional consequence of this difference, suggesting that this may be an example of developmental system drift (70) and that it may be a relatively neutral change. We suggest that together, these combined patterning mechanisms represent an ancestral insect eye ground plan that is deeply conserved. Despite this striking conservation of initial patterning, insects have found ways to diversify their eyes. We suggest that much of the observed diversity could result from modifications to developmental processes that occur after establishment of the ground plan itself.

Changes in TF expression could be responsible for eye patterning diversity. Modifications that repattern the eye could occur either through changes in how regulators and TFs known from *Drosophila* are expressed, or they could involve entirely different patterning factors. For example, in the evolution of nonstochastic patterning, the stochastic regulator Ss could be redeployed and expressed in new patterns. Perhaps mosquitos express Ss in deterministic ventral patterns. Unfortunately, neither butterfly nor *Drosophila* Ss antibodies cross-react across species and so this is left to be determined. It is also possible that the evolution of novel patterns occurred via expression of novel regulators, and this will be interesting to investigate further.

The ground plan is likely to include other deeply conserved factors. We discussed three steps in insect retina patterning. The first step, specification of eye vs. noneye, has been previously shown to be deeply conserved (3, 71), and we focus here on the third step of PR specification. It would also be interesting to examine the second step and determine whether earlier morphogenetic furrow signals are conserved across groups, such as the use of Decapentaplegic and Hedgehog signaling, as well as to compare expression and function of the genes that initiate furrow progression such as Eya. These could be additional components of the ground plan.

We showed examples where the ground plan itself has been modified in butterflies and honeybees by generating two R7s. It is interesting that this expansion in the number of ommatidial types and in the possible number of color receptor combinations occurred in groups where many species rely on colorful food sources and interact with colorful conspecifics – groups that contain the butterflies and the bees. In the future, it will be interesting to evaluate the genetic basis of how butterflies produce ommatidia that recruit two R7s.

The insect eye ground plan appears to be ancient and may extend beyond the insects. While crustacean PR number can vary, species in several groups have been shown to have eight, including mantis shrimp (Stomatopoda) and tadpole shrimp (Branchiopoda) (26, 72). Perhaps the most suggestive finding is that these eight PRs are recruited sequentially, in a similar pattern to the insects, in *Triops* (26), suggesting that this process may be very highly conserved and perhaps common to the Pancrustacea. It would be interesting to examine whether crustacean groups use the same TFs and signaling pathways and to determine where the ground plan arose.

While there is a rich historical literature on insect eye morphology and physiology that has identified many interesting features and various organizations of insect visual systems, a wealth of biodiversity remains yet to be characterized. New tools combined with the ever-improving availability of genome and transcriptome sequences provide an opportunity to explore the genetic basis of visual system adaptation.

## Materials and Methods

### Animal Husbandry.

The painted lady butterfly *Vanessa cardui*, the moth *Manduca sexta*, and the red flour beetle *Tribolium castaneum* were obtained from Carolina Biological Supply Company, California, USA. The fruit fly *Drosophila melanogaster*, the housefly *Musca domestica*, the honeybee *Apis mellifera*, and the mosquito *Aedes aegypti* and *C. quinquefasciatus* originated from laboratory stocks maintained at University of California San Diego. The house cricket *Acheta domesticus* were purchased from a local pet store in San Diego, California. The *Sphaerophoria* spp. hoverflies were captured as a male/female pair near Washington Square Park in New York, NY.

### Localization of Protein (Immunohistochemistry) and mRNA (HCR In Situ Hybridization).

The details of immunohistochemistry for embryonic, larval, pupal, adult retina were described in (36). Primary antibodies (36) were used with the following concentrations: rabbit anti-Sal (1:400), guinea pig anti-Dve (1:400), and rat anti-PxPros (1:100). AlexaFluor secondary antibodies were used as follows: donkey anti-rabbit-488 (1:250), donkey anti-guinea pig-555 (1:250), donkey anti-rat-647 (1:250), phalloidin (actin) (1: 250). In situ HCR 3.0 was performed as detailed in (73). Sequences used in the design of HCR probes are listed in SI Appendix, Table S1. HCR hybridization probes were designed as described in (74). Images of immunohistochemical stains and HCR in situ hybridizations were acquired using a Leica Sp8 confocal microscope.

### Functional Analysis of *sev* and *spi*.

We conducted CRISPR/Cas9 experiments to knock out *sev* in the butterfly *V. cardui* and the housefly *M. domestica*. We also knocked out *spi* in the cricket *A. domesticus* and the butterfly *V. cardui*. The details of butterfly embryo injection were described in (36), and are summarized here briefly: Butterfly eggs were collected from 1 h to 7 h after egg laying and injected with mixed of sgRNAs and Cas9 using borosilicate glass needles. One to two sgRNA were coinjected with yellow sgRNA for targeting y*ellow* color mutation. The final concentration of sgRNA/Cas9 was 250 ng/uL. After injection, the eggs were placed in petri dishes with dampened cotton to maintain humidity. After hatching, larvae were individually transferred to artificial diet in small 37 mL cups until pupation. The cricket eggs were collected from 6 h to 17 h after egg laying. The final concentration of sgRNA/Cas9 was 125~250 ng/uL. Housefly eggs were collected 30 min after egg laying, dechorionated with 25% bleach, and injected within 30 min with a mixture of concentration of sgRNA/Cas9 1,000 ng/µL. Synthetic sgRNAs were obtained from Synthego and specific target sequences can be found in SI Appendix, Table S2.

The expression of *sev* in the beetle *T. castaneum* was knocked down by using RNA interference (RNAi). DsRNA was injected into the abdomen of pupae with the concentration of 500 ng/uL. To synthesize dsRNA, target sites were chosen on exon 3, exon 7, and exon 8: Each target was amplified by PCR with primers containing the T7 promoter sequence as a 5’ overhang. The PCR product was purified using Zymo DNA clean and concentrator-5 kit and used as template for in vitro transcription using Invitrogen Megascript T7 transcription kit. DsRNA were purified using Zymo RNA clean and concentrator-100 kit, ethanol purified, and resuspended in water at a concentration of 150 ng/µL. DsRNA injection followed the standard protocol detailed in (75).

## Supplementary Material

Appendix 01 (PDF)

## Data Availability

All study data are included in the article and/or SI Appendix.

## References

[1] J.-Y. Roignant, J. E. Treisman, Pattern formation in the *Drosophila* eye disc. Int. J. Dev. Biol. **53**, 795–804 (2009). 19557685 10.1387/ijdb.072483jr PMC2713679

[2] J. Rister, C. Desplan, D. Vasiliauskas, Establishing and maintaining gene expression patterns: Insights from sensory receptor patterning. Development **140**, 493–503 (2013). 23293281 10.1242/dev.079095 PMC3561783

[3] R. Quiring, U. Walldorf, U. Kloter, W. J. Gehring, Homology of the eyeless gene of *Drosophila* to the small eye gene in mice and aniridia in humans. Science **265**, 785–789 (1994). 7914031 10.1126/science.7914031

[4] P. Callaerts, G. Halder, W. J. Gehring, PAX-6 in development and evolution. Annu. Rev. Neurosci. **20**, 483–532 (1997). 9056723 10.1146/annurev.neuro.20.1.483

[5] E. R. Vandendries, D. Johnson, R. Reinke, orthodenticle is required for photoreceptor cell development in the *Drosophila* eye. Dev. Biol. **173**, 243–255 (1996). 8575625 10.1006/dbio.1996.0020

[6] D. Arendt, Evolution of eyes and photoreceptor cell types. Int. J. Dev. Biol. **47**, 563–571 (2003). 14756332

[7] J. P. Kumar, My what big eyes you have: How the *Drosophila* retina grows. Dev. Neurobiol. **71**, 1133–1152 (2011). 21604387 10.1002/dneu.20921 PMC3212655

[8] J. Zhu, S. Palliyil, C. Ran, J. P. Kumar, *Drosophila* Pax6 promotes development of the entire eye-antennal disc, thereby ensuring proper adult head formation. Proc. Natl. Acad. Sci. U.S.A. **114**, 5846–5853 (2017). 28584125 10.1073/pnas.1610614114 PMC5468661

[9] B. P. Weasner, J. P. Kumar, The early history of the eye-antennal disc of *Drosophila melanogaster*. Genetics **221**, iyac041 (2022). 35460415 10.1093/genetics/iyac041 PMC9071535

[10] J. Warren, J. P. Kumar, Patterning of the *Drosophila* retina by the morphogenetic furrow. Front. Cell Dev. Biol. **11**, 1151348 (2023). 37091979 10.3389/fcell.2023.1151348 PMC10117938

[11] T. Wolff, D. F. Ready, The beginning of pattern formation in the *Drosophila* compound eye: The morphogenetic furrow and the second mitotic wave. Development **113**, 841–850 (1991). 1726564 10.1242/dev.113.3.841

[12] A. Tomlinson, D. F. Ready, Cell fate in the *Drosophila* ommatidium. Dev. Biol. **123**, 264–275 (1987). 17985474 10.1016/0012-1606(87)90448-9

[13] M. Domínguez, J. D. Wasserman, M. Freeman, Multiple functions of the EGF receptor in *Drosophila* eye development. Curr. Biol. **8**, 1039–1048 (1998). 9768358 10.1016/s0960-9822(98)70441-5

[14] M. Freeman, Reiterative use of the EGF receptor triggers differentiation of all cell types in the *Drosophila* eye. Cell **87**, 651–660 (1996). 8929534 10.1016/s0092-8674(00)81385-9

[15] A. Tomlinson, G. Struhl, Delta/notch and boss/sevenless signals act combinatorially to specify the *Drosophila* R7 photoreceptor. Mol. Cell **7**, 487–495 (2001). 11463374 10.1016/s1097-2765(01)00196-4

[16] A. Tomlinson, Y. E. Mavromatakis, G. Struhl, Three distinct roles for Notch in *Drosophila* R7 photoreceptor specification. PLoS Biol. **9**, e1001132 (2011). 21886484 10.1371/journal.pbio.1001132 PMC3160325

[17] R. Behnia, C. Desplan, Visual circuits in flies: Beginning to see the whole picture. Curr. Opin. Neurobiol. **34**, 125–132 (2015). 25881091 10.1016/j.conb.2015.03.010 PMC4577302

[18] C. Schnaitmann, M. Pagni, D. F. Reiff, Color vision in insects: Insights from *Drosophila*. J. Comp. Physiol. A **206**, 183–198 (2020). 10.1007/s00359-019-01397-3 PMC7069916 32020291

[19] S. Yamaguchi, R. Wolf, C. Desplan, M. Heisenberg, Motion vision is independent of color in *Drosophila*. Proc. Natl. Acad. Sci. U.S.A. **105**, 4910–4915 (2008). 18353989 10.1073/pnas.0711484105 PMC2290790

[20] T. Cook, F. Pichaud, R. Sonneville, D. Papatsenko, C. Desplan, Distinction between color photoreceptor cell fates is controlled by Prospero in *Drosophila*. Dev. Cell **4**, 853–864 (2003). 12791270 10.1016/s1534-5807(03)00156-4

[21] B. Mollereau , Two-step process for photoreceptor formation in *Drosophila*. Nature **412**, 911–913 (2001). 11528479 10.1038/35091076

[22] P. M. Domingos , Regulation of R7 and R8 differentiation by the spalt genes. Dev. Biol. **273**, 121–133 (2004). 15302602 10.1016/j.ydbio.2004.05.026

[23] R. J. Johnston , Interlocked feedforward loops control cell-type-specific rhodopsin expression in the *Drosophila* eye. Cell **145**, 956–968 (2011). 21663797 10.1016/j.cell.2011.05.003 PMC3117217

[24] M. F. Wernet , Stochastic spineless expression creates the retinal mosaic for colour vision. Nature **440**, 174–180 (2006). 16525464 10.1038/nature04615 PMC3826883

[25] J. Yan , Regulatory logic driving stable levels of defective proventriculus expression during terminal photoreceptor specification in flies. Development **144**, 844-855 (2017), 10.1242/dev.144030. 28126841 PMC5374349

[26] R. R. Melzer, C. Michalke, U. Smola, Walking on insect paths? Early ommatidial development in the compound eye of the ancestral crustacean *Triops cancriformis*. Naturwissenschaften **87**, 308–311 (2000). 11013878 10.1007/s001140050727

[27] M. Friedrich, Continuity versus split and reconstitution: Exploring the molecular developmental corollaries of insect eye primordium evolution. Dev. Biol. **299**, 310–329 (2006). 16973149 10.1016/j.ydbio.2006.08.027

[28] M. Friedrich, I. Rambold, R. R. Melzer, The early stages of ommatidial development in the flour beetle *Tribolium castaneum* (Coleoptera; Tenebrionidae). Dev. Genes Evol. **206**, 136–146 (1996). 24173466 10.1007/s004270050039

[29] D. F. Ready, A multifaceted approach to neural development. Trends Neurosci. **12**, 102–110 (1989). 2469216 10.1016/0166-2236(89)90166-5

[30] X. Yang, N. ZarinKamar, R. Bao, M. Friedrich, Probing the *Drosophila* retinal determination gene network in Tribolium (I): The early retinal genes dachshund, eyes absent and sine oculis. Dev. Biol. **333**, 202–214 (2009). 19324029 10.1016/j.ydbio.2009.02.040

[31] M. Friedrich, I. Rambold, R. R. Melzer, The early stages of ommatidial development in the flour beetle *Tribolium castaneum* (Coleoptera; Tenebrionidae). Dev. Genes Evol. **206**, 136–146 (1996). 24173466 10.1007/s004270050039

[32] B. Walcott, G. A. Horridge, The compound eye of Archichauliodes (Megaloptera). Proc. R. Soc. Lond. B Biol. Sci. **179**, 65–72 (1971).

[33] H. Ohuchi, T. Bando, T. Mito, S. Noji, Eye Development and Photoreception of A Hemimetabolous Insect, Gryllus Bimaculatus in The Cricket As A Model Organism. (Springer, Japan, 2017), pp. 49–62.

[34] Y. Dong, L. Dinan, M. Friedrich, The effect of manipulating ecdysteroid signaling on embryonic eye development in the locust *Schistocerca americana*. Dev. Genes Evol. **213**, 587–600 (2003). 14618403 10.1007/s00427-003-0367-z

[35] M. F. Wernet, M. W. Perry, C. Desplan, The evolutionary diversity of insect retinal mosaics: Common design principles and emerging molecular logic. Trends Genet. **31**, 316–328 (2015). 26025917 10.1016/j.tig.2015.04.006 PMC4458154

[36] M. Perry , Molecular logic behind the three-way stochastic choices that expand butterfly colour vision. Nature **535**, 280–284 (2016). 27383790 10.1038/nature18616 PMC4988338

[37] L. Lichtenstein, K. Grübel, J. Spaethe, Opsin expression patterns coincide with photoreceptor development during pupal development in the honey bee. *Apis mellifera*. BMC Dev. Biol. **18**, 1 (2018). 29382313 10.1186/s12861-018-0162-8 PMC5791347

[38] X. Liang, S. Mahato, C. Hemmerich, A. C. Zelhof, Two temporal functions of glass: Ommatidium patterning and photoreceptor differentiation. Dev. Biol. **414**, 4–20 (2016). 27105580 10.1016/j.ydbio.2016.04.012 PMC4875859

[39] S. Mahato , Common transcriptional mechanisms for visual photoreceptor cell differentiation among pancrustaceans. PLoS Genet. **10**, e1004484 (2014). 24991928 10.1371/journal.pgen.1004484 PMC4084641

[40] F. Zufall, M. Schmitt, R. Menzel, Spectral and polarized light sensitivity of photoreceptors in the compound eye of the cricket (*Gryllus bimaculatus*). J. Comp. Physiol. A **164**, 597–608 (1989). 2709343 10.1007/BF00614502

[41] R. H. White, H. Xu, T. A. Münch, R. R. Bennett, E. A. Grable, The retina of *Manduca sexta*: Rhodopsin expression, the mosaic of green-, blue- and UV-sensitive photoreceptors, and regional specialization. J. Exp. Biol. **206**, 3337–3348 (2003). 12939366 10.1242/jeb.00571

[42] Y. E. Mavromatakis, A. Tomlinson, R7 photoreceptor specification in the developing *Drosophila* eye: The role of the transcription factor Deadpan. PLoS Genet. **12**, e1006159 (2016). 27427987 10.1371/journal.pgen.1006159 PMC4948816

[43] T. G. W. Graham, S. M. A. Tabei, A. R. Dinner, I. Rebay, Modeling bistable cell-fate choices in the *Drosophila* eye: Qualitative and quantitative perspectives. Development **137**, 2265–2278 (2010). 20570936 10.1242/dev.044826 PMC2889600

[44] A. Tomlinson, D. F. Ready, Sevenless: A cell-specific homeotic mutation of the *Drosophila* eye. Science **231**, 400–402 (1986). 17735014 10.1126/science.231.4736.400

[45] A. Tomlinson, Y. E. Mavromatakis, R. Arias, The role of Sevenless in *Drosophila* R7 photoreceptor specification. Dev. Biol. **454**, 181–189 (2019). 31207209 10.1016/j.ydbio.2019.06.007 PMC6726512

[46] M. Tio, K. Moses, The *Drosophila* TGFα homolog Spitz acts in photoreceptor recruitment in the developing retina. Development **124**, 343–351 (1997). 9053310 10.1242/dev.124.2.343

[47] M. Freeman, The spitz gene is required for photoreceptor determination in the *Drosophila* eye where it interacts with the EGF receptor. Mech. Dev. **48**, 25–33 (1994). 7833286 10.1016/0925-4773(94)90003-5

[48] K. E. Brown, M. Kerr, M. Freeman, The EGFR ligands Spitz and Keren act cooperatively in the *Drosophila* eye. Dev. Biol. **307**, 105–113 (2007). 17512517 10.1016/j.ydbio.2007.04.025

[49] B. Misof , Phylogenomics resolves the timing and pattern of insect evolution. Science **346**, 763–767 (2014). 25378627 10.1126/science.1257570

[50] M. J. Henze, K. Dannenhauer, M. Kohler, T. Labhart, M. Gesemann, Opsin evolution and expression in arthropod compound eyes and ocelli: Insights from the cricket *Gryllus bimaculatus*. BMC Evol. Biol. **12**, 163 (2012). 22935102 10.1186/1471-2148-12-163 PMC3502269

[51] X. Hu , Patterned rhodopsin expression in R7 photoreceptors of mosquito retina: Implications for species-specific behavior. J. Comp. Neurol. **516**, 334–342 (2009). 19637310 10.1002/cne.22114 PMC2845463

[52] R. C. Hardie, Functional Organization of The Fly Retina in Progress in Sensory Physiology (Springer, 1985), pp. 1–79.

[53] Q.-X. Chen, B.-Z. Hua, Ultrastructure and morphology of compound eyes of the scorpionfly *Panorpa dubia* (Insecta: Mecoptera: Panorpidae). PLoS One **11**, e0156970 (2016). 27258365 10.1371/journal.pone.0156970 PMC4892548

[54] W. A. Ribi, J. Zeil, The visual system of the Australian ‘Redeye’ cicada (Psaltoda moerens). Arthropod Struct. Dev. **44**, 574–586 (2015). 26335848 10.1016/j.asd.2015.08.005

[55] S. Laughlin, S. McGinness, The structures of dorsal and ventral regions of a dragonfly retina. Cell Tissue Res. **188**, 427-447 (1978). 647758 10.1007/BF00219782

[56] M. Friedrich, E. J. Wood, M. Wu, Developmental evolution of the insect retina: Insights from standardized numbering of homologous photoreceptors. J. Exp. Zool. B Mol. Dev. Evol. **316B**, 484–499 (2011). 10.1002/jez.b.21424 21796775

[57] K. Kral, Ultraviolet vision in European owlflies (Neuroptera: Ascalaphidae): a critical review. Eur. J. Entomol. **99**, 1–4 (2002).

[58] B. S. Wells, D. Pistillo, E. Barnhart, C. Desplan, Parallel Activin and BMP signaling coordinates R7/R8 photoreceptor subtype pairing in the stochastic *Drosophila* retina. eLife **6**, e25301 (2017). 28853393 10.7554/eLife.25301 PMC5599232

[59] A. C. Miller, E. A. Urban, E. L. Lyons, T. G. Herman, R. J. Johnston, Interdependent regulation of stereotyped and stochastic photoreceptor fates in the fly eye. Dev. Biol. **471**, 89–96 (2021). 33333066 10.1016/j.ydbio.2020.12.008 PMC7856283

[60] K. Arikawa, The eyes and vision of butterflies. J. Physiol. **595**, 5457–5464 (2017). 28332207 10.1113/JP273917 PMC5556174

[61] O. Trujillo-Cenóz, G. D. Bernard, Some aspects of the retinal organization of Sympycnus lineatus loew (Diptera, dolichopodidae). J. Ultrastruct. Res. **38**, 149–160 (1972). 5009747 10.1016/s0022-5320(72)90089-5

[62] H. Ebadi , Patterning the insect eye: From stochastic to deterministic mechanisms. PLoS Comput. Biol. **14**, e1006363 (2018). 30439954 10.1371/journal.pcbi.1006363 PMC6264902

[63] F. Schmeling , Opsin expression, physiological characterization and identification of photoreceptor cells in the dorsal rim area and main retina of the desert locust. *Schistocerca gregaria*. J. Exp. Biol., **217**, 3557-3568 (2014), 10.1242/jeb.108514. 25104757

[64] M. W. Perry, C. Desplan, Love spots. Curr. Biol. **26**, R484–R485 (2016). 27326705 10.1016/j.cub.2016.02.020 PMC5154687

[65] M. P. Sison-Mangus, G. D. Bernard, J. Lampel, A. D. Briscoe, Beauty in the eye of the beholder: The two blue opsins of lycaenid butterflies and the opsin gene-driven evolution of sexually dimorphic eyes. J. Exp. Biol. **209**, 3079–3090 (2006). 16888057 10.1242/jeb.02360

[66] J. G. Menzel, H. Wunderer, D. G. Stavenga, Functional morphology of the divided compound eye of the honeybee drone (*Apis mellifera*). Tissue Cell **23**, 525–535 (1991). 18621175 10.1016/0040-8166(91)90010-q

[67] G. A. Horridge, M. McLean, The dorsal eye of the mayfly Atalophlebia (Ephemeroptera). Proc. R. Soc. Lond. B Biol. Sci. **200**, 137–150 (1978).

[68] M. A. Jervis, A taxonomic revision of the pipunculid fly genus *Chalarus* Walker, with particular reference to the European fauna. Zool. J. Linn. Soc. **105**, 243–352 (1992).

[69] M. F. Land, Visual acuity in insects. Annu. Rev. Entomol. **42**, 147–177 (1997). 15012311 10.1146/annurev.ento.42.1.147

[70] J. R. True, E. S. Haag, Developmental system drift and flexibility in evolutionary trajectories. Evol. Dev. **3**, 109–119 (2001). 11341673 10.1046/j.1525-142x.2001.003002109.x

[71] P. Callaerts, J. Clements, C. Francis, K. Hens, Pax6 and eye development in Arthropoda. Arthropod Struct. Dev. **35**, 379–391 (2006). 18089082 10.1016/j.asd.2006.09.002

[72] M. L. Porter, H. Awata, M. J. Bok, T. W. Cronin, Exceptional diversity of opsin expression patterns in *Neogonodactylus oerstedii* (Stomatopoda) retinas. Proc. Natl. Acad. Sci. U.S.A. **117**, 8948–8957 (2020). 32241889 10.1073/pnas.1917303117 PMC7183149

[73] H. M. T. Choi , Third-generation in situ hybridization chain reaction: Multiplexed, quantitative, sensitive, versatile, robust. Development **145**, dev165753 (2018). 29945988 10.1242/dev.165753 PMC6031405

[74] E. Kuehn , Segment number threshold determines juvenile onset of germline cluster expansion in *Platynereis dumerilii*. J. Exp. Zool. B Mol. Dev. Evol. **338**, 225–240 (2022). 34793615 10.1002/jez.b.23100 PMC9114164

[75] N. Posnien RNAi in the red flour beetle (*Tribolium*). Cold Spring Harb. Protoc. **8**, prot5256 (2009). 10.1101/pdb.prot5256 20147232

