# Supplementary material for: Deep conservation complemented by novelty and innovation in the insect eye ground plan
Source: Proc Natl Acad Sci U S A. 2024 Dec 30;122(1):e2416562122. doi: 10.1073/pnas.2416562122 (PMC11725883; doi:10.1073/pnas.2416562122)
Supplement: Supplementary file 1 — Appendix 01 (PDF) [file pnas.2416562122.sapp.pdf]

Supporting information for: Deep conservation complemented by novelty and innovation in the insect eye ground plan

Figure S1

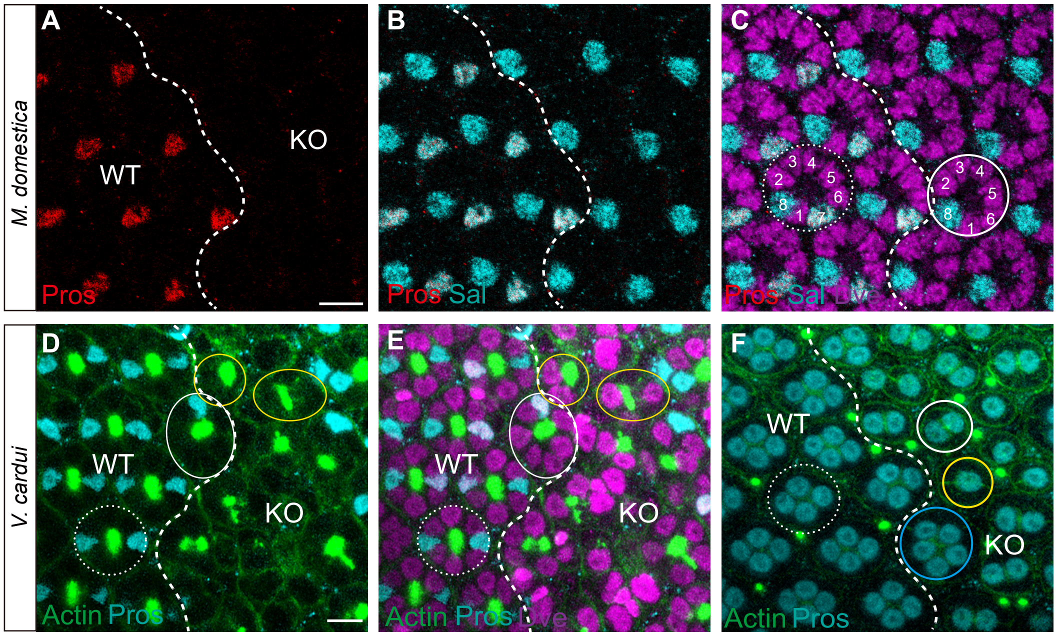

Figure S1. The effect of Sevenless loss-of-function on PR recruitment in the house fly M. domestica and butterfly V. cardui.

(A-C) CRISPR-induced mosaic knockout of sev results in the loss of R7 cells in the pupal retina of M. domestica. (A) Disrupted regions in sev KO pupal retinas lack Pros-positive (red) R7 PR nuclei, which are visible in adjacent wild type (WT) regions. Approximate clone boundaries are indicated by a white dashed line. (B) In WT regions, Sal staining (cyan) is observed in R7 and R8, but only the cell in the R8 position remains in disrupted sev KO regions (B). Outer PRs R1-6 are marked by Dve expression (magenta) in both WT and KO regions (C). Scale bar, 10 µm.

(D, E) CRISPR-induced mosaic knockout of sev results in the loss of R7 and four outer PRs in pupal retinas of V. cardui. (D) In WT regions, two R7s per ommatidium are stained with Pros (cyan) and six outer PRs are stained with Dve (magenta). In KO regions, the absence of Pros-marked nuclei indicates the loss of both R7s. (E) Outer PRs are missing in sev KO regions. Yellow solid circles highlight examples of ommatidia with four missing outer PRs. The white solid circle highlights an ommatidium near the clone boundary that has lost only one R7, indicating that Sev is directly required in R7 recruitment. Dashed circle shows example of an ommatidium in a WT region, for comparison. Scale bar, 10 µm.

(F) In a different focal plane from the same confocal stack as (D-E), ommatidia in WT regions contain four cone cells per ommatidium, as labeled by Pros (cyan). Missing cone cells are observed in sev KO regions. This could indicate a possible role for Sev in cone cell recruitment, but more likely results from the lower number of outer PRs, which normally use EGFR/Spitz signaling to recruit cone cells. Fewer outer PRs could result in fewer cone cell recruitment events. The white solid circle highlights an ommatidium with two missing cone cells, a yellow solid circle shows an example with three missing cone cells, and the blue solid circle shows an example where ommatidia adjacent to WT regions sometimes gain additional cone cells. A dashed circle shows an example ommatidium in the WT region. PR cells are outlined by actin staining (green). The approximate boundary between WT and KO regions is indicated by a white dashed line. WT, wild type; KO, knockout.

Figure S2

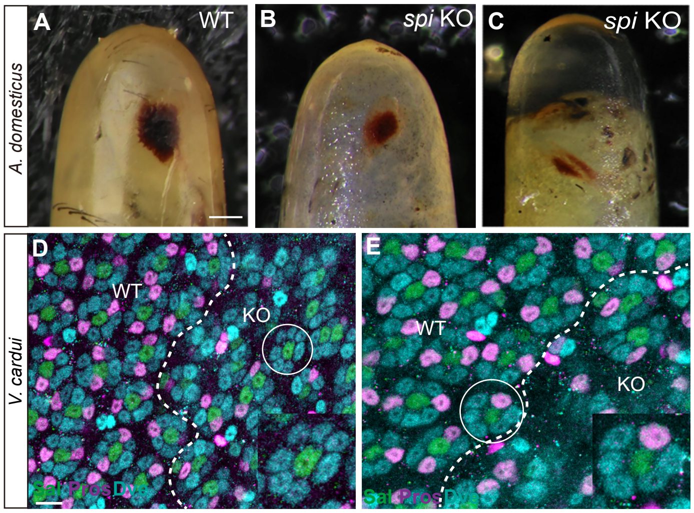

Figure S2. The effect of Spitz loss-of-function on PR recruitment in the cricket A. domesticus and butterfly V. cardui. (A) Anterior WT A. domesticus egg with developing retinas visible (red). Scale Bar, 100 µm. (B,C) Anterior spi KO A. domesticus eggs showing retinas of reduced size. (D,E) In V. cardui butterflies, CRISPR-induced mosaic knockout of spi results in the loss of R7 cells as well as outer PRs. In WT regions of pupal retinas, Pros (magenta) is expressed in two R7s and Sal (green) is expressed in R8 and the two Pros-marked R7s per ommatidium. In KO regions we observed a range of phenotypes. In (D), a white circle highlights an ommatidium that have lost both R7s (also see inset). Scale bar 10 µm. In (E), a white circle highlights an ommatidium that has lost one R7 and one outer PR (also see inset). Sal (green) is expressed in R8 in both WT and KO region. Outer PRs are stained with Dve (cyan). The boundary between WT and KO regions is shown by a white dashed line. WT, wild type; KO, knockout.

Figure S3

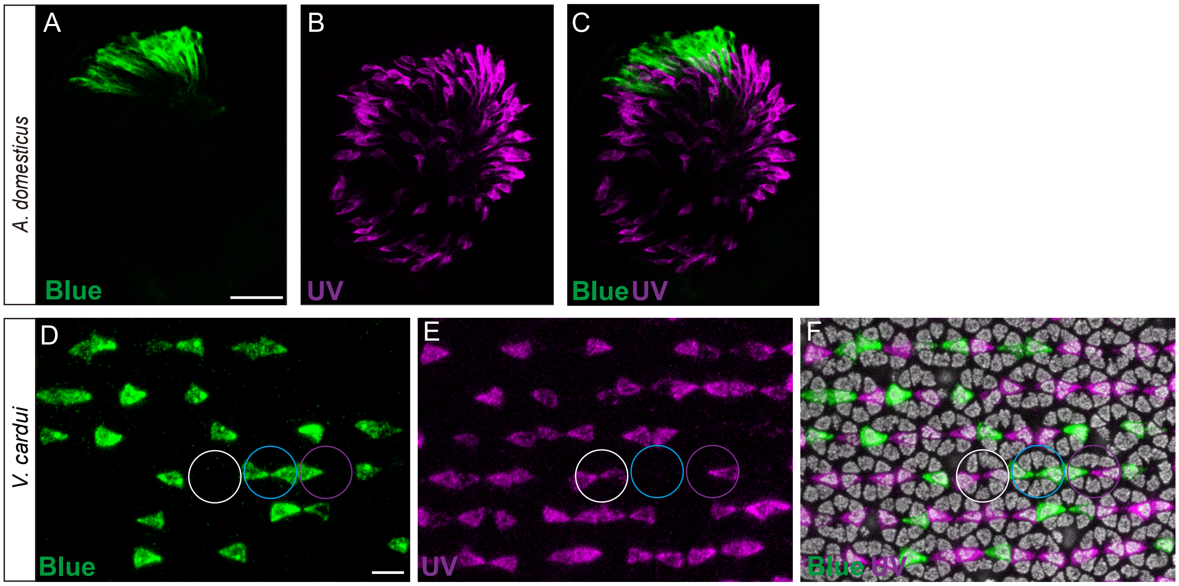

Figure S3. HCR in situ hybridizations visualize Rh expression. (A-C) In a newly hatched cricket nymph of A. domesticus, blue-sensitive Rh expression in a dorsal rim area while UV-sensitive Rh is expressed in regions outside the DRA, with no ventral region that expresses the Blue-sensitive Rh yet visible. Scale bar, 50 µm. (D-F) In V. cardui butterflies, UV and Blue-sensitive Rhs are stochastically expressed in R7 PRs. Ommatidia of type I express Blue-Blue (white circle), type II express UV-UV(blue circle), and type III express Blue-UV (magenta circle) in pupal retinas. Scale bar, 10 µm.

Table S1 Sequences used to design HCR probes.

Species	Opsins	Sequences
Acheta domesticus	UV	MLYVAFCGIALVGNGLVIWVFSSAKSLRTPSNVFVINLAICDFIMMLKTPIFIYNSF<br>NLGFAMGQLGCQIFAFMGSISGIGAAITNACIAYDRYRVIARPFDSKMSIKGATM<br>LVLLVWAYTLPWAIMPLLEVWGRFAPEGYLSSCSFDYLTDTPENHMFVLCIFICSY<br>VIPMSLIIYFYSQIVSHVVNHEKSLKEQAKKMNVDSLRSNQQQNQTSAEIRIAKVA<br>IGICFLFVASWTPYAVLALIGAFGNKTLLTPGVTMIPACTCKAVACLDPYVYAISHP<br>RYRVELQKRLPWLCIKEQTASDASSVATTTSTNATTTTTT
	Blue	MNSTTSFDGAPIALPYESYVVQLLGWNIPAEHIELVHPHWRGYETPSKFWHFGF<br>AFMYFCIMVMSCLGNGIVLWIFGTTKSLRTPSNMFVVNQALLDMLMMIEMPLF<br>VLNSLFYQRPIGWEVGCDIYALLGSVSGIGSAINNAAIAYDRYRTIAFPLDGRLQF<br>GHAMAFIFGTWVWAMPFSLLPLLRVWGRYVPEGFLTTCSFDYLTDDEDTRVFT<br>ASIFVWSYAFPLCLIIFFYCKLFNQVRFHEKMLKEQARKMNVKSLQTNQDAEQKS<br>VEIRIAKVAFTIFFLFLCSWTPYATVAMIGAFGNRALLTPMSTMIPALTAKIVSCID<br>PWVYAINHPRFRGELLRRAPWFGVKEINPSDVGSVTTDRTTTTGTTEAVSA
Vanessa cardui	UV	MIPVLNMDNKTENYNIYGAYFAPLRSSDGIKMLVDGLEGEDLAAVPEHWFSYAA<br>PPASAHTALALLYCFFTAAALIGNGLVVFIFATTKSLRTSSNLLILNLAIFDFIMMAK<br>APLFIYNSAMRGFATGALGCQIFAVMGSYSGIGAGMTNACIAYDRHSTITRPLDG<br>RLSRGKALLMIALVWIYATPWSLMPLFKVWGRFVPEGYLTSCTFDYLSNTFDTKL<br>FVACIFVCSYVFPMSFIIYFYSGIVKQVFAHEAALREQAKKMNVESLRSNQNASAE<br>SAEIRIAKAALTVCFLFVASWTPYGVMSLIGAFGDQQLLTPGVTMIPAVTCKLVA<br>CIDPWVYAISHPKYRQELQRRMPWLQINEPDDNASTGTNNTANSSAPATA
	Blue	MATNYTDDIGPVAYPLKMVTQEVVEHMLGWNIPEDHQDLVHEHWRNFPAVS<br>KYWHYGLAFIYTILMLASVSGNGIVIWIFSTSKSLRSASNMFVINLAVFDLMMML<br>EMPMLVVNSFYQRLLGYQLGCDIYAVLGSLSGIGGAMTNAIIAFDRYKTISSPLDG<br>RLNRVQASLLILFSWLWALPFTFLPAFRVWGRYVPEGFLTTCSFDYMTDDQDTKI<br>FVMCIFVWSYVIPMTFICCFYSKLFGAVRLHERMLKEQAKKMNVKSLAANKEDS<br>GKSIEIRIAKVAFTIFFLFLCSWTPYAFVTMTGAFGDRGLLTPVATMVPAVCAKIVS<br>CIDPWVYAINHPRYRAELQKRLPWMGVREADPDSVSSASGATAQTQNPTAEA
Culex quinquefasciatus	UV	MPFLEHLSDNYTAVLRPEARLSAETRYLGWNVAPEDLPHIPEHWLKYPEPEASLH<br>YLLGLLYIAFTIFALVGNGLVIWIFSSAKSLRTPSNVFIVNLAICDFLMMVKTPIFIYN<br>SFTRGFTTGYLGCQIFAFIGSLSGIGAGATNACIAYDRYNTIARPFEGKLTHTKAIFI<br>TCLVWAYTIPWGLLPLMEIWGRFVPEGYLTSCTFDYLTNTFDNRMFVGTIFTFSY<br>VLPMSLIIYYYSQIVSHVVNHEKALREQAKKMNVDSLRSNQNQANSSVEVRIAKA<br>AITVCFLFVASWTPYAVLALIGAFGDKSLLTPGVTMFPACACKFVACLDPYVYAIS<br>HPRYRVELQKRLPWLAITETLPSDTASTTTEATTTNATASS
	Blue	MFLLNETDAVLLPAARTGGEMVKLLGWNLPPEQMHLVHEHWKDFPAPPYFM<br>HLLLALIYFVLMNVSLIGNGIVVWIFTTSKSLRNGSNMFIVNLAIFDLLMMCEMP<br>MFLVNAFSERLVGYETGCAIYAALGSVSGIGGAISNAVIAYDRYRTISNPLEGRMN<br>RTKASLFVVMTWLWTVPFTVMPMFNIWGRYIPEGYLTTCSFDYLTDDSDTRVF<br>VGCIFAWAYAIPMVFICYFYTRLFGHVRQHENMLKNQARKMNIESLAANRNAN<br>AEAAEIRIAKAAFTIFFLFVCAWTPYAFVAMIGAFGDKTILTPFFTMIPAMCCKIVS<br>CLDPWVYAISHPRYRQELEKRLPWLGIKEAPDNVSTTESKQTVVAEPAANAET

Table S2 sgRNA guides used in this study.

Species	Genes	CRISPR sgRNA	Sequences
Vanessa cardui	*Sevenless*	VcSev1	GGCAAGTTCTAGTCGCACTG
Vanessa cardui	*Spitz*	VcSpi1	GGGAAGGTGACATTCAGGCG
Vanessa cardui	*Spitz*	VcSpi2	AGAGTGGATACGTGGGCCCG
Vanessa cardui	*Spitz*	VcSpi3	CCAACGACTGCATTGTCCGC
Acheta domesticus	*Spitz*	AdSpi1	CAGUACCAGGUGGCGUAAGC
Acheta domesticus	*Spitz*	AdSpi2	CACAGUCCUCGGCAUCGCGG
Musca domestica	*Sevenless*	Mdsev1	GGGCGACAGGTCATTATCGG
Musca domestica	*Sevenless*	Mdsev2	CCGCCACCGCCAATTCTGGC
Vanessa cardui	*Yellow*	VcY2	GGCCTTATCGCTTACTCCT
